# Bacteria communities and water quality parameters in riverine water and sediments near wastewater discharges

**DOI:** 10.1038/s41597-022-01686-8

**Published:** 2022-09-21

**Authors:** Carolina Oliveira de Santana, Pieter Spealman, Daniella Azulai, Mary Reid, M. Elias Dueker, Gabriel G. Perron

**Affiliations:** 1 Geosciences Institute, Federal University of Bahia, Salvador, BA 40170-290 Brazil; 2 Center for Genomics and Systems Biology, New York University, New York, NY 10114 USA; 3 Department of Biology, Reem-Kayden Center for Science and Computation, Bard College, Annandale-On-Hudson, NY 12504 USA; 4 Bard Center for Environmental Sciences and Humanities, Bard College, Annandale-On-Hudson, NY 12504 USA

**Keywords:** Water microbiology, Environmental impact

## Abstract

Wastewater treatment plant (WWTP) discharges alter water quality and microbial communities by introducing human-associated bacteria in the environment and by altering microbial communities. To fully understand this impact, it is crucial to study whether WWTP discharges affect water and sediments microbial communities in comparable ways and whether such effects depend on specific environmental variables. Here, we present a dataset investigating the impact of a WWTP on water quality and bacterial communities by comparing samples collected directly from the WWTP outflow to surface waters and sediments at two sites above and two sites below it over a period of five months. When possible, we measured five physicochemical variables (e.g., temperature, turbidity, conductivity, dissolved oxygen, and salinity), four bioindicators (e.g., *Escherichia coli*, total coliforms, *Enterococcus* sp., and endotoxins), and two molecular indicators (e.g., *intI1*’s relative abundance, and *16S rRNA* gene profiling). Preliminary results suggest that bioindicators correlate with environmental variables and that bacterial communities present in the water tables, sediments, and treated water differ greatly in composition and structure.

## Abstract (summary)

**Table:** 

Measurement(s)	temperature of water • conductivity of water • dissolved oxygen in water • salinity of water • Concentration of Escherichia coli in water • Concentration of total coliforms in water • Concentration of Enterococcus sp. • Concentration of endotoxins in water • Relative abundance of integron 1 in water • Bacterial 16S RNA
Technology Type(s)	YSI field probe • Colilert dection system • Enterolert dection system • Charles River Endosafe system • quantitative PCR • Illumina Sequencing
Sample Characteristic - Organism	Bacteria
Sample Characteristic - Environment	Fresh water river
Sample Characteristic - Location	United States

## Background & Summary

The discharge of effluents from wastewater treatment plants (WWTPs) into surrounding waterways can have detrimental effects on the health of aquatic ecosystems. For one, WWTPs discharge important pollutants, including pharmaceuticals^1,2^ and household products^3^, which can impact on the local fauna^4^ as well as microbial communities^5,6^. WWTP can also be a source of allochthonous microorganisms distinct from the receiving waterway, including pathogens^7^ and antibiotic-resistant bacteria^8^. Even when most live microorganisms are eradicated by WWTP treatment, WWTPs have been recognized as an important source of antibiotic resistance genes^9,10^, contributing to the ever-growing reservoir of antibiotic resistance in the environment^11^. WWTP discharges can also lead to nutrient enrichment^12^, which can cause significant changes in detectable dissolved oxygen^13^ and disrupt biotic community structure and function in aquatic environments^14^. Finally, WWTP discharge can deposit sand and grit into aquatic systems, affecting the physical characteristics of sediment and potentially disrupting sediment-associated bacterial communities found in waterways^15^.

**Fig. 1:**
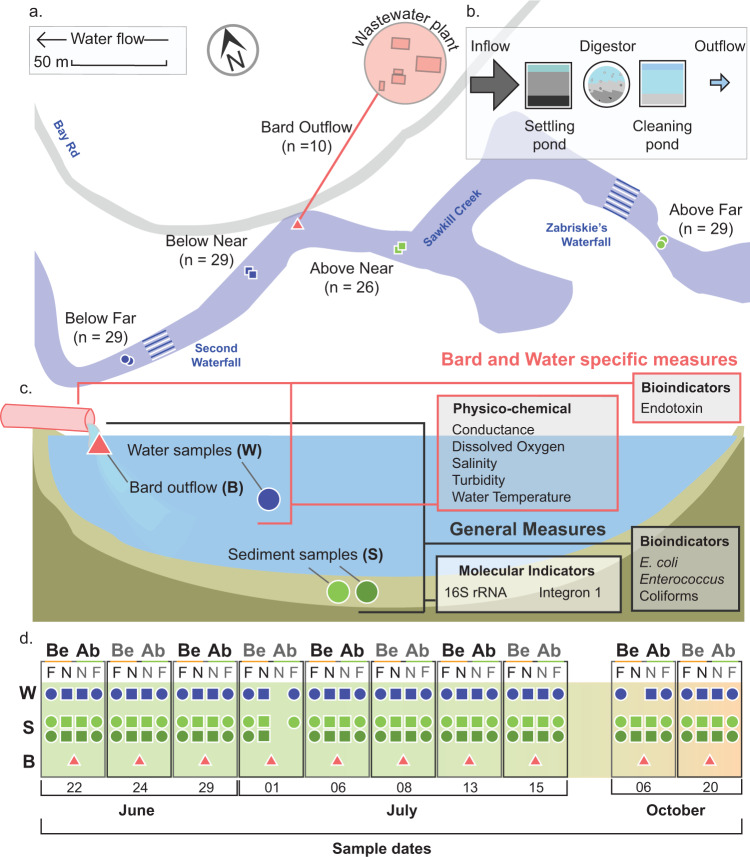
Diagram of study. Representative map of sampling sites used in this research with site names, total sample number, and GPS coordinates (**a**) Schematic representation of the WWTP included in this study (**b**) Schematic representation of sampling types (Bard outflow (B); Water (W); Sediment (S)) and the sampling measures performed, including those applied to all samples (General Measures) and those specific to Bard and Water sample sites (**c**) Lastly, a timeline of 10 sampling events over 5 months shows the number of successful samples taken from the W, S, and B sample types for both the Below (Be) and Above (Ab) and at the Far (F) and Near (N) sites (**d**).

To assess the possible impacts of small WWTPs on local freshwater waterways, we monitored microbial contaminants relating to the treated water outflow of the WWTP operated by Bard College (Annandale-on-Hudson, NY; Fig. 1a). Using a digester-based system (Fig. 1b), the WWTP treats an estimated 0.2 million gallons per day (per NY SPDES permit #NY0031925). This treated water is produced entirely by the Bard College campus, a residential college serving approximately 2,000 students and 500 faculty and staff. After treatment, used water is released into the Saw Kill, a tributary of the Hudson River, which is also the source of freshwater for the campus. Bard College pulls an average of 130,000 gallons (591,000 L) per day of Saw Kill surface waters for campus drinking water (as per the most recent report filed with the NYSDEC Bureau of Water Resource Management, dated 2/9/21). The drinking water is withdrawn from the Saw Kill about 100 m upstream from the WWTP outflow. The research site itself is located approximately 5.2 km downstream from the Village of Red Hook (pop. ~ 1900), which also has a small WWTP (NY SPDES permit #NY0271420).

To investigate the possible impacts of the Bard College WWTP on bacterial communities found in the surrounding environments, we collected samples from the treated used waters directly from the outflow as well as from surface waters and sediments at two sites above and below the outflow (Fig. 1c) over a period of five months (Fig. 1d). Here, we provide an overview of the sample collection and data collection, without detailed analysis of results or discussion, to draw attention to its comprehensive and multi-faceted view into freshwater microbial communities in relation to physicochemical and microbial indicators of water quality. In addition, the consistency in experimental design, sequencing methodology, and sample sources ensures the value of this collection for ongoing studies of freshwater microbial communities, particularly those pertaining to temporal, spatial, and anthropogenic disturbance.

**Fig. 2:**
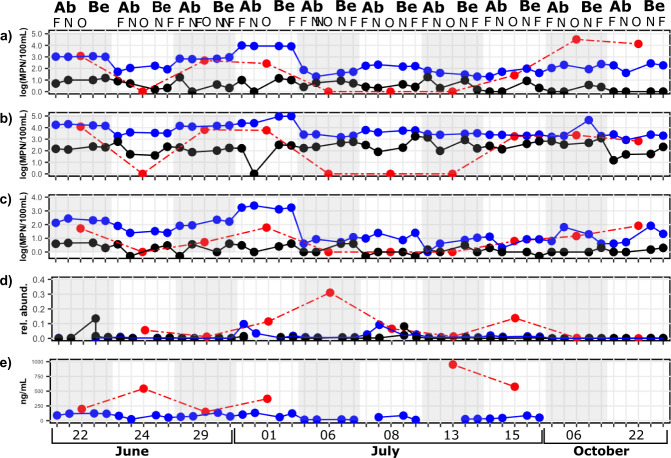
Longitudinal measurements of microbial indicators of water quality. For each collection date, samples were taken at four different sites and from the Bard Water treatment plant outflow and were evaluated for: (**a**) *E. coli* concentration; (**b**) Coliform concentration; (**c**) *Enterococcus* sp. concentration; (**d**) integron 1 relative abundance; and (**e**) endotoxin concentration. Outflow and water data points depict the raw value for each sample while sediments data points are the average of two biological replicates. Surface water samples are presented in blue, sediments in black and outflow in red.

More specifically, for each water sample, including the outflow, we measured various physicochemical characteristics: temperature, turbidity, conductivity, dissolved oxygen, and salinity. We also assessed sewage impact on these samples using three contamination indicators: *Escherichia coli* concentration, total coliforms concentration, *Enterococcus* sp. concentration. We also estimated endotoxin concentrations and the presence of the *intI1* gene, a marker of integron 1, abundance relative to the abundance of the *16S rRNA gene*, a marker of the total bacterial abundance. Interestingly, while we observed that all microbiological indicators follow similar temporal trends (Fig. 2), we found different levels of correlation between the microbiological indicators (Table 1), suggesting that each indicator might provide unique insights on the ecology of microbial contaminants in the studied system.

**Table 1:** Coefficients of correlation between microbiological indicators.

	*IntI1*	*E. coli*	Coliforms
*E. coli*	0.4	—	
Coliforms	0.2	0.7	—
*Enterococcus sp.*	0.4	0.8	0.6

**Fig. 3:**
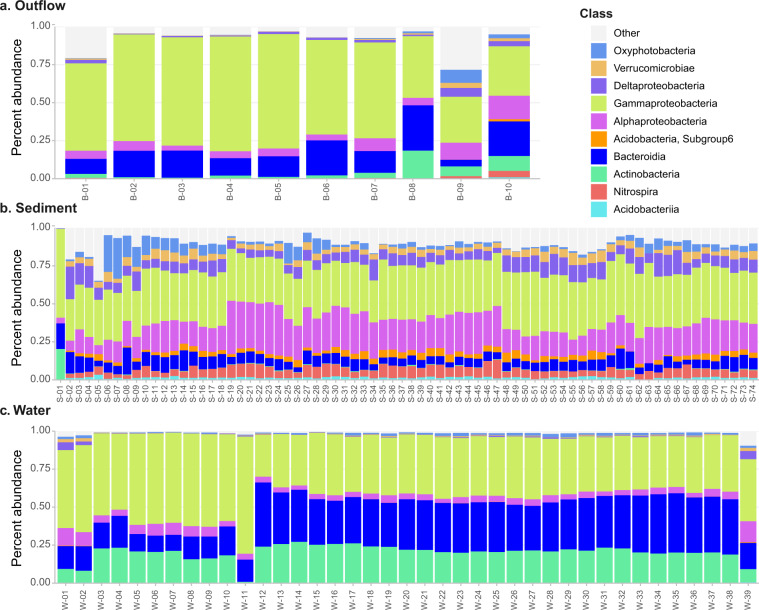
Relative frequency of prokaryotic taxa at the level of class isolated from samples of (**a**) outflow, (**b**) sediments, and (**c**) water. The 10 most abundant classes and their prevalence in each sample type are shown. The details regarding specific sites and dates of collection for each sample are shown in “Sample_type_date_site_season_name.csv” available at Dryad^20^. It can be observed that the outflow samples generally present lower diversity at the Class level when compared to the water samples and the sediment samples, which in turn are the most diverse in this dataset.

Finally, we characterized the bacterial population present in each sample using *16S rRNA* amplicon sequencing. Overall, the sequencing generated 3,020,375 paired-reads with a median of 24,556 reads per library. After removing chimeric, mitochondrial and chloroplast sequences, the 3,019,951 (99.98% of original) paired sequences were assigned to 15,140 amplicon sequence variants, or ASVs, belonging to 723 genera. Interestingly, we found that prokaryotic classes differed in distribution and abundance between the water, sediment, and outflow samples and, to a lesser extent, between the different sites sampled (Fig. 3).

To our knowledge, this study is one of the first to simultaneously consider multiple microbial contaminants as well as molecular contaminants^16^ in both water and sediment microbial communities in an aquatic environment^17–19^. Not only will this study shed a new light on the limited understanding of the complexities related to extra-enteric sewage indicator ecology and management^7^, but also will provide a unique dataset to explore on the dynamic of microbial contaminants in relation to microbial community structures in freshwater ecosystems.

## Methods

### Study site

The Saw Kill is a 23.0 km tributary of the Hudson River that rises in the town of Milan (41.985169, −73.776175) and drains a 57 km^2^ area of northwestern Dutchess County, New York. The Saw Kill flows predominantly through forests and farmland and flows at a mean rate of 0.54 m^3^ s^−1^ (ranging from 0.01–9.15 m^3^ s^−1^) into the South Tivoli Bay (42.02061, −73.92367), located between Montgomery Place and Bard College (flow data from USGS StreamStats, USGS Station # 01364800, measurements made in 1965). More recent but unpublished measurements have been made on the waterway as part of the Saw Kill Monitoring Project: monthly measurements taken between November 2017 and November 2018 at the Lower Saw Kill Dam (located within 10 m of our above-near site) using an AVG flow monitor estimated a mean flow rate of 0.97 +/− 0.22 m^3^ s^−1^, confirming that the flow rate range was consistent over the past decades. While the Bays are separated from the Hudson River by a built embankment, the latter empties into the Hudson River via two artificial channels (North Bridge: 42.045689, −73.924926, South Bridge: 42.036672, −73.925465) built by Amtrak in 1850.

The Saw Kill serves as the primary source of drinking water for the Bard College Campus located in Annandale-On-Hudson. To this end, Bard College contains an insular water system for its campus, bringing potable water to its buildings through a filter-based drinking water system and disposing of wastewater via its wastewater treatment plant downstream of the drinking water intake. For treating wastewater, the Bard water treatment plant operates by filtration, sedimentations, fermentation in a bioreactor network, and chlorination. The treated wastewater is then sent down for aeration followed by de-chlorination before being released into the Saw Kill via a single outflow pipe at a site approved by the New York State Department of Environment Conservation (SPDES Permit # NY0031925) (Fig. 1b). The duration of the overall process depends on the volume of wastewater treated and environmental conditions. The treated wastewater is finally released in an area located near the mouth of the Saw Kill in a predominantly wooded area.

It is important to note that the Saw Kill has other possible sources for both fecal indicators and associated bacterial signals upstream of Bard’s campus, including Red Hook (5.2 km upstream), a village (pop. ~1900) with a small WWTP (7.6 × 10^5^ L ٠ day^−1^ flow, NY SPDES permit #NY0271420). Furthermore, the intervening land use includes rural and exurban habitation with aging septic systems and agricultural land use. As such, our goal was to isolate the localized influence of the Bard College WWTP as part of the larger Saw Kill Watershed system and the final allochthonous source to the tributary before it meets the tidal Hudson River. To achieve this, we sampled both upstream (Fig. 1: Above) and downstream (Fig. 1: Below) of the Bard WWTP outflow (Fig. 1: Outflow).

### Sample collection

Samples were collected on ten different occasions over a period of five months ranging from June 6, 2015, and October 20, 2015 (Sampling_site_metadata_table.csv available at Dryad^20^; Fig. 1d). For each collection date, we collected treated wastewater directly from the WWTP outflow, from two sites below the outflow, and two sites above the outflow (estimated GPS coordinates for each site can be found in “Sample_site_GPS.csv” available at Dryad^20^. Because we were sampling (and therefore disturbing) stream sediments as part of this study, sampling began with the most downstream site and we worked upstream, ensuring that any disturbances of sediments would not be reflected in subsequent samples taken that day. In addition to the 2 L wastewater sample from the outflow, we collected 2 L of surface water and two ~500 mg sediment cores at each collection site. In total, we thus collected 130 samples, including 40 surface water samples, 80 sediment samples, and ten wastewater samples from the WWTP outflow (“Sampling_type__date_site_season_name.csv” available at Dryad^20^.

We first collected the 2 L water sample mid-channel for each site using heat and acid-sterilized Nalgene bottles submerged ~0.5 m below the stream’s surface. To avoid possible contamination that could be present on the surface of the bottles, all sample bottles were rinsed three times with surface waters from the site immediately before collection. After collecting the water sample, sediment samples were collected using a stainless-steel corer, which was cleaned with a wipe, sterilized with 70% ethanol, and air-dried in between each sampling. At each site, duplicate cores about 7 cm deep were collected from undisturbed sediment and placed in a sterile 50 ml falcon tube using a sterilized metal spatula. Finally, for each collection date, a single 2 L samples from the WWTP were taken from the end of the outflow pipe using a sterilized 1,000-ml scoop and stored in heat and acid-sterilized Nalgene bottles. Following collection, all samples were placed on ice in a cooler for transport to the lab and processed within 2 hours of collection.

### Nucleic acid extraction

We extracted total DNA from water samples by filtering 750 mL of water onto a 0.22 µm Sterivex filter. We then extracted DNA from the filter using the PowerWater DNA Isolation kit (MoBio Laboratories Carlsbad, CA, USA), now available as the DNeasy PowerWater DNA Isolation Kit (QIAGEN, Hilden, Germany), per the manufacturer’s instructions. As for sediment samples, we weighed 250 mg of sediment samples and used the PowerSoil DNA Isolation Kit (MoBio Laboratories, Carlsbad, CA, USA), now available as the DNeasy PowerSoil DNA Isolation kit (QIAGEN, Hilden, Germany) as the per manufacturer’s instructions.

### Physico-chemical water quality indicators

At each sampling site, before collecting water and sediment samples, we measured water temperature (°C), conductivity (µmhos/cm), dissolved oxygen (ppm), and salinity (ppm) using handheld YSI field probes (YSI, TN, USA) with the probes suspended at <0.5 m depth midchannel. Turbidity was measured in the lab using 15 mL aliquots from shaken 2 L sample bottles with a Hach 2100 P Turbidimeter (Loveland, CO) and all data is recorded in “Physicochemical_characteristics.csv” available at Dryad^20^.

### Meteorological variables

Air temperature at the time of sampling was recorded from the Albany, NY, office of the National Weather Service. Rain precipitation amounts (mm) in the 12, 24, 38, 48, and 72 hours prior to sampling were also gathered from the Albany, NY, office of the National Weather Service. All data is available in “Sampling_site_metadata_table.csv” available at Dryad^20^.

### Concentrations of microbial water quality indicators

In each sample, we measured the abundance of three water quality indicators: *Enterococcus*, *Escherichia coli*, and Total Coliforms using EPA-approved standard methods IDEXX MPN methods (IDEXX, Westbrook, ME, USA; 40 CFR 141.852(a)(5)). As per manufacturer instructions, all three indicators were assayed within 2 hours of sample collection in the field. To run the IDEXX Colilert assay, which estimates both *E. coli* and coliform concentrations, on mid-channel water samples, a 100 mL undiluted sample and one 100 mL sample diluted 1:10 with sterile DI water were assayed. For the WWTP outflow samples, a 1:10 and 1:100 dilution with sterile DI water were assayed. For sediments, slurries were prepared by adding 250 mg of centrifuged sediment to 50 mL of sterile DI water and mixing gently and we assayed a 1:10 and a 1:100 dilution of the sediment slurry^21^. For each sample assayed, Colilert reagents were dissolved in the sample in a sterile 100 mL vial. Once dissolved, the mixture was poured into a 49-well sterile Quanti-Tray (IDEXX, Westbrook, ME, USA) and sealed. The trays were then incubated for 24 hours at 35 °C. Following incubation, the Quanti-Tray are enumerated for positive counts where all cells that have turned yellow are considered positive for coliform, and all yellow cells that fluoresce under UV excitation are considered positive for *E. coli*. The concentrations of *E. coli* and Coliforms indicators are then calculated as MPN/100 mL by applying the Most Probable Number (MPN) method to the number of positive cells and are found in “Escherichia_coli_concentration.csv” and “Total_coliforms_concentration.csv” respectively and available at Dryad^20^.

To estimate the concentration of *Enterococcus* sp. in each sample, we used the IDEXX Enterolert assay (IDEXX, Westbrook, ME, USA). For surface water samples and outflow samples, we assayed 100 mL of an undiluted sample while we used undilute slurry (see above for details) for sediment samples. Enterolert reagents were dissolved in the 100 mL sample in a sterile 100 mL vial. Once dissolved, the mixture was poured into a 49-well sterile Quanti-Tray (IDEXX, Westbrook, ME, USA) and sealed. The trays were then incubated for 24 hours at 41 °C. After incubation, all cells that fluoresce under UV light are considered positive and used to estimate the concentration of the contaminants as MPN/100 mL and is stored in “Enterococcus_concentration.csv” available at Dryad^20^.

### Concentration of endotoxins

Endotoxins were measured in water samples within 4 hours of sampling using the Charles River Endosafe system (Charles River, Cambridge, MA, USA) with cartridges supporting a 10-0.1 EU/mL measurement range. Before measurement, using endotoxin-free pipette tips, 20 μL of water sample was diluted with 1980 μL of sterile, endotoxin-free Hyclone water in a sterile and endotoxin-free glass test tube to create a 1:100 dilution. As per Charles River’s Endosafe protocol, once a new sterile cartridge was validated by the Endosafe system, 25 μL of the sample were then pipetted into each of the four cartridge wells without introducing bubbles. These wells represented duplicate raw readings and duplicate spike readings (for detection of endotoxin enhancement or inhibition by sample content). Once the aliquots were in place, the assay was begun, taking 5–15 minutes of testing time before displaying data. Readings that were fully validated by the instrument (those whose spike returns were between 50 and 200% and whose replicate variations (both sample and spike) had a coefficient of variation <25%) were recorded. Invalid tests prompted a second assay, using a 1:1000 dilution to dilute contaminants and/or bring the sample into the measurement range. This data is recorded in “Endotoxins_concentration.csv” available at Dryad^20^.

### Relative abundance of integron 1

Following the methodology described elsewhere^22^ and using the primers listed here^23^, we processed each sample in triplicate using the PowerUp SYBR Green Master Mix (Applied Biosystems, Foster City, CA, USA) and using the Bio-Rad CFX96 Real-Time PCR Detection System (Bio-Rad Laboratories, Hercules, CA, USA). Next, we built an internal standard curve for each run using at least three dilutions of the strain *Escherichia coli* SK4903 with IncPβ R751, which was constructed to contain seven *16S rRNA* copies and six *intI1* copies. Finally, we adjusted the total number of *16S rRNA* copies found in each sample by dividing that number by 4.2, which is the average number of *16S rRNA* copies each bacteria cell harbors^24^. This data is found in “Integron_1_relative_abundance.csv” available at Dryad^20^.

### Amplification of *16S rRNA* sequences and analysis

A 16S rRNA gene amplicon sequencing library targeting the V4 region was amplified using primers 515 F and 806 R as described in the Earth Microbiome Project^25^. Samples were shipped to Wright Labs (Huntingdon, PA, USA) for sequencing performed with the Illumina Miseq platform using 250-bp paired ends. Sequences were filtered and trimmed with Trimmomatic, ver. 0.39^26^, using the following parameters: ILLUMINACLIP:TruSeq3-PE.fa:2:30:10 LEADING:3 TRAILING:3

SLIDINGWINDOW:4:15 MINLEN:100. All subsequent analysis was performed using QIIME2, ver 2019.2^27^. Reads were resolved, denoised, and clustered into amplicon sequence variants (ASVs) using DADA2 (denoisepaired, --p-trim-left-f 13, --p-trim-left-r 13, --p-trunc-len-f 150, --p-trunc-len-r 130)^28^ the results of this are available in “Denoising_qc_stats.tsv”, available at Dryad^20^. Reads per sample are recorded in “Sample_frequency_detail.csv”, available at Dryad^20^. Taxonomic assignment was performed using QIIME2’s naive Bayes scikit-learn classifier^29^ trained using the 16S rRNA gene sequences in SILVA database (Silva SSU 132)^30^. Taxonomic abundance per sample is recorded in “Taxa_abundance_by_sample.csv”, available at Dryad^20^ while ASV, taxa assignment, and confidence are recorded in “ASV_and_taxa_assignment.tsv”, available at Dryad^20^. Data visualization was performed using phyloseq (ver. 3.14)^31^, ggplot2 (ver. 3.35)^32^, and fantaxtic (ver. 0.1.0, G. Martijn).

## Data Records

All data and outputs of taxonomic have been deposited within the Dryad repository^20^ and are listed in Table 2. Raw data of 16S rRNA amplicon sequencing (fastq file) have been deposited with links to BioProject accession number PRJNA565393 in the NCBI BioProject database^33^.

**Table 2:** List of data and materials available with this study.

File Name	Description
Sampling_site_metadata_table.csv	Comma separated file containing a list of sampled sites with date, cumulative rainfall (mm) and air temperature (°C)
Physicochemical_characteristics.csv	Comma separated file containing listing water Temperature (°C), Turbidity (TU), Conductivity (µmhos/cm), Dissolved Oxygen (mgL), and Salinity (ppt) for all sample
Escherichia_coli_concentration.csv	Comma separated file containing concentration of *Escherichi coli* (MPN·100 mL^−1^) for all samples
Total_coliforms_concentration.csv	Comma separated file containing concentration of Coliform (MPN·100 mL^−1^) for all samples
Enterococcus_sp_concentration.csv	Comma separated file containing concentration of *Enterococcus* sp. (MPN·100 mL^−1^) for all samples
Endotoxins_concentation.csv	Comma separated file containing concentration of Endotoxin (EU·mL^−1^) for all samples
Integron_1_relative_abundance.csv	Comma separated file containing abundance of *intI1* relative to *16S rRNA* for all samples
ASV_and_taxa_assignment.tsv	Tab separated file containing taxa assignment and percent confidence for each ASV
Taxa_abundance_by_sample.csv	Comma separated file listing all taxa at species level resolution (or lowest possible) and abundances in each sample
Sawkill_mapping_and_env_var.csv	Comma separated file containing sample names, sample sites and measured environmental variables.
Denoising_qc_stats.tsv	Tab-separated file containing ‘input’, filtered’, ‘denoised’, ‘merged’, and ‘non-chimeric’ read abundances per sample.
Sample-frequency-detail.csv	Comma separated file containing final reads used per sample
Sample_site_GPS.txt	Text file containing map-refined estimated GPS coordinates (decimal format) of all sample sites.
Sample_type_date_site_season_name.csv	Comma separated file containing Sample, type, date, site and season as used to generate Fig. 3.

## Technical Validation

The handheld YSI Probe was calibrated before fieldwork using standard protocols. Briefly, the DO probe was placed in a 100% humid environment by moistening a sponge and placing it in the calibration sleeve, which was then placed over the probe for 10 minutes before conducting the automated calibration process. Salinity and conductivity were calibrated using a fresh YSI-provided conductivity calibration solution (used within one month of opening the bottle). Enterolert and Colilert assays were conducted with 2 DI water controls, incubated, and enumerated to validate sterile technique. Along with the rigorous internal controls of the Endosafe instrument, a control sample using the sterile endotoxin-free water was run during each Endosafe session. To control for possible microbial and DNA contamination during DNA extraction, we included three different types of controls at each sampling point: 1) sterivex filtration using sterile PCR-grade water to test for contamination during filtration of samples; 2) DNA extraction conducted on PCR-grade water to test for contamination due to the DNA extraction kits; and 3) library construction conducted on sterile PCR-grade water ot test for possible contamination due to PCR reagents. In all cases, we failed to detect the presence of contamination during library construction and sequencing. To control for possible biases between runs of qPCR, an internal standard curve was constructed using at least four dilutions of genomic DNA of *E. coli* strain SK4903. In addition, all PCRs were performed in triplicates, and multiple negative controls (PCR reaction without template) were interspaced between DNA samples during the PCR preparation and the amplification. The success of 16S rRNA gene amplicon generation was controlled by reviewing the amplicon size (approximately 291 bp) and absence of contaminations on an agarose gel. Negative (PCR reaction without template) and positive controls (genomic DNA of *E. coli* DH5a) also ensured purity of the employed reagents.

## Data Availability

No custom code was used to generate or process these data. All commands for QIIME2, phyloseq, ggplot2, and fantaxtic are available in r-markdown format in a single file named “Combined_Scripts.rmd” in the Dryad repository^20^.
